# Pupillary dilations in a Target/Distractor visual task paradigm and attention deficit hyperactivity disorder (ADHD)

**DOI:** 10.1016/j.neulet.2023.137556

**Published:** 2023-11-10

**Authors:** Claudio M Privitera, Sean Noah, Thom Carney, Stanley A Klein, Agatha Lenartowicz, Stephen P Hinshaw, James T McCracken, Joel T Nigg, Sarah L Karalunas, Rory C. Reid, Mercedes T Oliva, Samantha S Betts, Gregory V Simpson

**Affiliations:** a School of Optometry and Vision Science, University of California, Berkeley, United States; b Department of Psychiatry & Biobehavioral Sciences, University of California, Los Angeles, United States; c Department of Psychology, University of California, Berkeley, Department of Psychiatry and Behavioral Sciences, University of California, San Francisco, United States; d Department of Psychiatry and Biobehavioral Sciences, Semel Institute and David Geffen School of Medicine, University of California, Los Angeles, United States; e Departments of Psychiatry and Behavioral Neuroscience, Oregon Health & Science University, Portland, United States; f Department of Psychological Sciences, Purdue University, West Lafayette, IN, United States; g Division of Social Sciences, University of California, Santa Cruz, United States; h Graduate Program in Neurosciences, University of Southern California, Los Angeles, United States; i Think Now, Incorporated, San Francisco, CA, United States; j Helen Wills Neuroscience Institute, University of California, Berkeley, United States

## Abstract

ADHD is a neurocognitive disorder characterized by attention difficulties, hyperactivity, and impulsivity, often persisting into adulthood with substantial personal and societal consequences. Despite the importance of neurophysiological assessment and treatment monitoring tests, their availability outside of research settings remains limited. Cognitive neuroscience investigations have identified distinct components associated with ADHD, including deficits in sustained attention, inefficient enhancement of attended Targets, and altered suppression of ignored Distractors. In this study, we examined pupil activity in control and ADHD subjects during a sustained visual attention task specifically designed to evaluate the mechanisms underlying Target enhancement and Distractor suppression. Our findings revealed some distinguishing factors between the two groups which we discuss in light of their neurobiological implications.

## 1. Introduction

Attention Deficit/Hyperactivity Disorder (ADHD) is a neurocognitive disorder characterized by behaviors like difficulty sustaining attention, restlessness, and impulsivity. People with ADHD may struggle to focus, exhibit levels of agitation, and often act impulsively. It is usually diagnosed in childhood using a set of criteria based on observed behavior and reported symptoms, but it can remain undetected and persistent until adulthood[7,38,2]. Adult ADHD is prevalent and enduring with substantial personal and societal impacts but objective methods for characterization of the disorder are limited [17].

Cognitive neuroscience studies have identified two functionally and neuronally distinct components of selective attentional control that can be altered independently: (a) a top-down controlled enhancement of attended targets and (b) suppression of ignored distractors[8,35,49]. ADHD symptoms include distractibility to irrelevant stimuli and difficulty to protect the ongoing focus of attention from distractors[37,1].

Pupil activity and attention are closely interconnected. In visual search, for example, we have found that pupillary dilation indicates target detection and decision-making processes[32,33]. A wide volume of data show how this dilation mechanism expands to many other cognitive tasks and can index cognitive workload, performance, and attentional effort in general[14,16,18,39,20,45,21].

We measured pupil activity of participants with ADHD and neuro-typical controls in a sustained visual attention experiment specifically designed to assess the aforementioned principle of attending-Targets vs. ignoring-Distractors enhancement/suppression mechanism.

## 2. Methods

We enrolled 76 adults: 42 Control and 34 ADHD (age range 18–40 years old) in the Berkeley, University of California, site. All participants in the ADHD sample were recruited from the University clinics, pools from prior University ADHD studies, local clinics, private practice clinicians and ADHD groups. Neurotypical controls were volunteers that responded to a participants recruitment flyer.

Specific inclusion criteria for ADHD participants included the following: (i) meeting of DSM-5 criteria for ADHD (American Psychiatric Association, [2]) presenting with clinically significant levels of impairment as assessed by structured clinical interview and the Adult ADHD Clinical Diagnostic Scale (ACDS DSM-5); (ii) Clinical Global Impression-Severity (CGI-S) as indicated by a score ≥ 4; (iii) estimated IQ equal or greater than 80 (measured by WASI-II); (iv) allowance of anxiety disorders, persistent depressive disorder (dysthymia), antisocial personality disorder, oppositional defiant disorder (ODD), conduct disorder (CD), nicotine abuse or dependence allowed as comorbidities for the ADHD sample—to enhance generalizability; (v) ability to complete required study procedures.

Exclusion criteria (assessed with the Mini International Neuropsychiatric Interviews, MINI, DSM-V) for ADHD participants were as follows: lifetime history of bipolar disorder, major depression disorder (MDD), psychotic disorder, pervasive developmental disorder, substance abuse, or substance dependence (except nicotine). We excluded MDD while keeping Persistent Depressive Disorder (PDD) because MDD involves being in episode, while PDD is often considered a syndrome marked by lower levels of symptom severity without a clear episodic course. We felt that current MDD would interfere too much with their cognitive testing, thereby confounding the study data and assessment.

Exclusion criteria for ADHD and neurotypical participants included the following: (i) history of childhood neurodevelopmental disorder other than ADHD (e.g., autism, dyslexia); (ii) history of a general medical condition requiring chronic use of medication with CNS effects on cognitive performance; (iii) history of seizure disorder, brain tumor, other major neurological disorder, or head injury resulting in loss of consciousness; (iv) history of serious oxygen deprivation; (v) current psychopathology requiring ongoing treatment with antipsychotic medications, mood stabilizers, benzodiazepines, or anticonvulsants; (vi) current untreated psychopathology rated as greater in severity than ADHD per se; and (vii) current treatment with guanfacine (because of the unacceptable risks of rapid withdrawal). Neurotypical control participants were matched to the ADHD sample for age, gender, estimated IQ (IQ > 80 measured by WASI-II), and years of education.

One of the challenges in identifying a clinically generalizable sample of adults with ADHD is the well-known rates of psychiatric comorbidity, especially for mood and anxiety disorders. We are unaware of conclusive data suggesting that maintenance treatment with antidepressants for mood or anxiety syndromes significantly confounds cognitive testing. We thus excluded any medications with known cognitive effects (atypical antidepressants, antipsychotics, mood stabilizers, benzodiazepines, anticonvulsants) while allowing participants with stable maintenance treatment with antidepressants (stable dose for greater than 3 months) to be enrolled. We do not have the data for how many participants were taking typical antidepressants. To minimize acute effects of stimulants and atomoxetine on cognitive performance, participants were asked to refrain from ADHD medication use for a period of approximately 24 h prior to testing sessions. Such an approach was justified by the best available data showing limited durations of behavioral and performance effects beyond 12––14 h for stimulants and atomoxetine [25,47].

All participants were invited to come into the University testing facility for a diagnostic assessment lasting 1–2 h. Those participants who passed the screening and were diagnosed and assigned to the ADHD or Control group were then invited to come for a second and then a third visit one month later, each lasting about 1 h, for our experimental protocol.

Participants performed the experiments in scotopic condition in a booth of approximately 2×2 square meters that isolated them from the surrounding laboratory. The booth contained a chair and a small desk with the stimulus monitor as the only source of illumination, along with the pupil-tracker apparatus: EyeLink 1000 head supported eye-tracker system (https://www.sr-research.com/EL_1000.html).

**Fig. 1.**
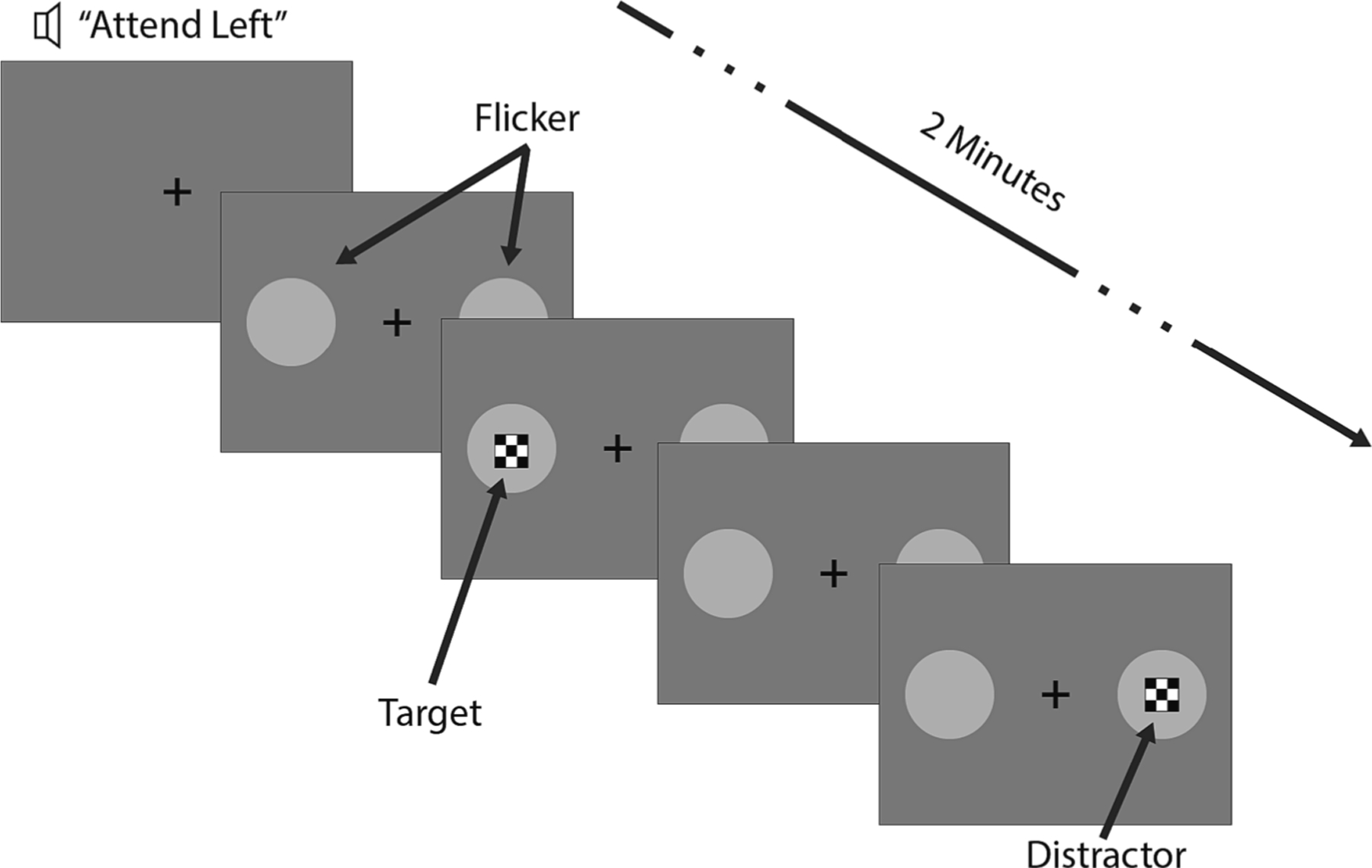
Targets and Distractors took the form of a square checkerboard superimposed over two circular light gray patches flickering on a darker gray background. A black cross was positioned at the center of the two gray patches for eye fixation. An audio message just prior the start of the experiment would define the side to attend (left in this example). Targets and Distractors appeared every 1–5 s (randomized continuously) and remained on for 100 ms.

The visual settings of the experiment consisted of two circular light gray patches of 1.5 deg in diameter and located 12 deg apart horizontally, flickering on a darker gray background (Fig. 1). A black cross was positioned at the center of the two gray patches for eye fixation. The frequency of the flickering was well beyond the 2 Hz break frequency of the pupil servomechanism and thus could not influence low-frequency pupil fluctuations[31]. Targets and Distractors took the form of a square checkerboard superimposed over the flicker region, with side lengths of 1 deg. The checkerboard pattern consisted of four smaller squares spatially alternating between black and the gray used in the flicker patches. Targets and Distractors appeared every 1–5 s (randomized continuously) and remained on for 100 ms. The sequences for the two patches were independent, so that there was not any temporal perceptual relationship between the stimuli at the two locations. An a udio message saying “left” or “right” (of the fixation point) and just prior to the start of the experiment defined the side to attend. The participant was instructed to respond with a button press to target (a patch in the attended side) detection and to ignore patches that occurred on the non-attended side (i.e. distractors). Each trial of stimulus events lasted two minutes and included an average of forty patch stimulus presentations drawn on top of the flickering patch (Fig. 1, see Target or Distractor); trials were then repeated seven or eight times with a few minutes break in between. The entire task lasted approximately 45 min, with each trial being unique, as the sequence and timing of Targets and Distractors were randomized for each iteration.

**Fig. 2.**
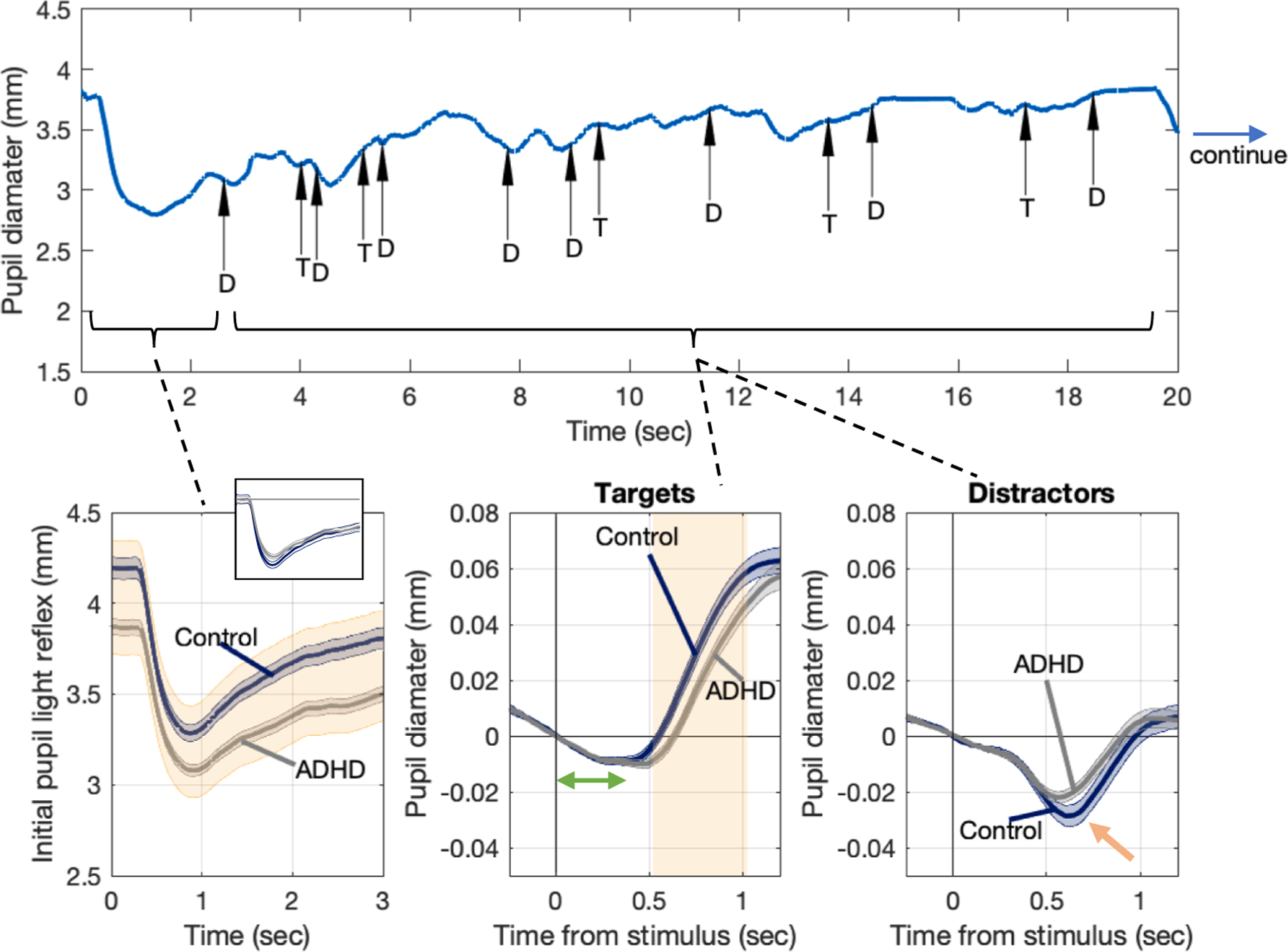
Top Inset. Pupil diameter was recorded for two minutes but only the first 20 s are displayed in this example (top inset). Arrows show the moment of presentation of Target (T) and Distractors (D). Bottom left panel. A light-induced pupil constriction characterizes the first three seconds of each trial; solid lines show the average waveform of Control (blue) and ADHD (gray), standard error is indicated by a shaded area of the same color. Areas of statistical difference between waveforms (for p < 0.01) are represented by the orange shaded background – when shifted vertically for comparison (small inset) the shape of the two average waveforms coincide. Bottom middle and right panel. Pupil segments are extracted from the following 117 s of the trial, grouped, averaged, and displayed based on Targets vs. Distractors and Controls vs. ADHD; time zero (and diameter zero) indicates the time of (and the pupil diameter at) the visual stimulus presentation. Controls have a more rapid onset of the dilation for Target and a larger constriction for Targets.

Pupil diameter was recorded at a sampling frequency of 1000 Hz for the duration of the measurement (the example in Fig. 2 top panel shows only the first 20 s of one trial of one participant) and subsequently segmented into short periods of 1.5 s corresponding to each of the visual stimuli, with time zero (and diameter zero) indicating the time of (and the diameter at) stimulus presentation. These segments were finally grouped based on the stimulus (Targets or Distractors) and population (Controls or ADHD). Their average waveform is reported with a solid line in blue for Controls and gray for ADHD, with standard error indicated by a shaded area of the same color (Fig. 2, lower, middle and right panels, standard error refers to the mean of the mean of the waveforms of each subject). For significance testing between groups, we used the same cluster-based permutation test used and discussed in previous works[24], see also, [32]; and [10]. According to the test, areas of statistical significance between waveforms (for p < 0.01) are represented by an orange-shaded background.

Eye blink artifacts were detected off-line in post-processing analysis using the information included in the Eyelink eye tracker output and recovered with a linear fit. Each individual trial was visually inspected to check the presence of major tracking artifacts and, in case, removed from the analysis, about 25 % of the total number of trials.

## 3. Results

Each trial was analyzed into two parts. The initial three-seconds (Fig. 2, bottom-left panel) reveal a rapid constriction of the pupil in response to the presentation of the gray background on the stimulus monitor at the beginning of the trial. Controls showed larger pupil diameters at baseline and through the entire duration of the reflex (Fig. 2, bottom-left panel), but with the same waveform as that of ADHD participants (small inset in Fig. 2, bottom-left panel, see mean waveforms shifted vertically for comparison and well overlapped).

**Fig. 3.**
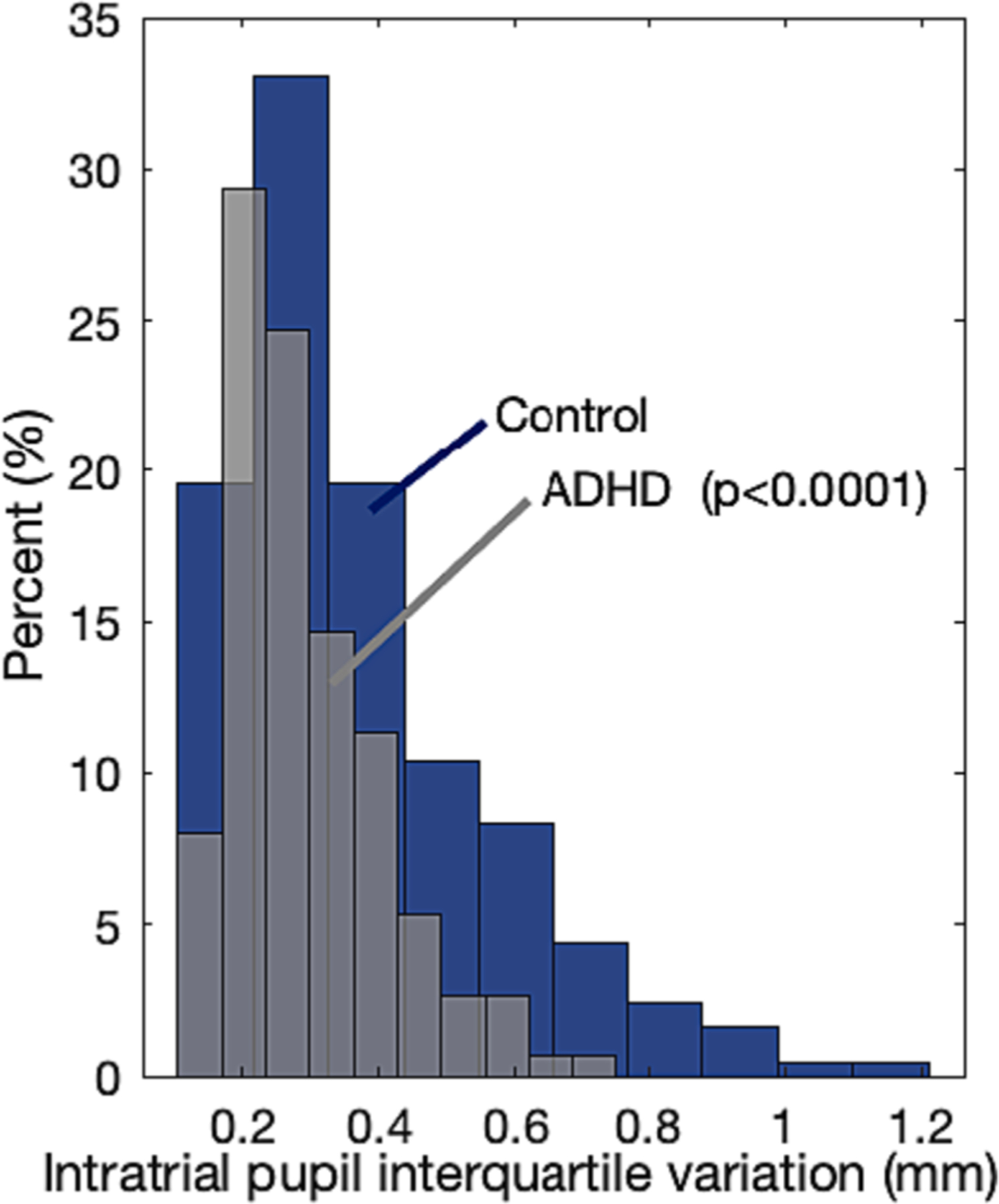
Pupil variability during the 117 s of the trial corresponding to the actual visual experiment is measured by the difference between the 75th and the 25th percentiles (interquartile distance) and is higher in Controls.

The following 117 s of the trial corresponding to the actual visual experiment are characterized by a low-frequency quasi-erratic pattern of dilation-recovery oscillations (Fig. 2, top panel) resembling pupil unrest [40]. We measured the amount of this activity using the interquartile distance, a measure of dispersion or variability given by the difference between the 75th and the 25th percentiles of the pupil diameters during the entire recording. It was larger for Controls (Fig. 3), 0.42 vs. 0.33, p < 0.0001, per an independent-sample *t*-test with a Cohen’s d effect size of 0.44. A different approach based on the analysis of the power spectrum of the Fourier transform in the low-frequency bandwidth of 0.1–2 Hz (see also [27]yielded the same conclusions: Controls have larger variations within the frequency range of interest.

Pupil segments are grouped, averaged, and displayed based on the stimulus, as well as Targets vs. Distractors and Controls vs. ADHD, (Fig. 2, bottom, middle and right panel). Time zero (and diameter zero) indicates the time of (and the pupil diameter at) the visual stimulus presentation. Both Targets and Distractors show an initial period of latency lasting approximately 250 msec after the presentation (green double arrow), during which the waveforms almost perfectly overlap, and the pupil does not yet show any significant reaction. This latent period is then followed by two different behaviors: a dilation for Targets (Fig. 2, bottom, middle panel) and a constriction for Distractors (Fig. 2, bottom, right panel). In response to Targets, onset and rate of the dilation is quicker for Controls than for participants with ADHD (Fig. 2, bottom, middle panel). For Distractors, the overall waveform of the constriction does not show a significant difference as measured by the cluster-based permutation criterion. However, when considering only the peak time of the constriction (indicated by the orange arrow in Fig. 2, bottom right panel), the amplitude of that constriction is significantly larger for Controls (0.015 mm vs 0.23 mm, p < 0.0001) according to an independent-sample *t*-test with a Cohen’s d effect size greater than 2.

## 4. Discussion

### 4.1. Phasic and tonic pupil behavior in the experiment

We conducted a simple visual experiment to assess sustained attention in a Target vs. Distractor paradigm consisting of a fixation point and small visual stimuli appearing intermittently on the right and left inside of the fixation. Subjects were asked to attend only one side (Target) while ignoring the other (Distractor) and to report Target detection with a button press. Controls have larger tonic pupils at baseline and through the entire course of the pupil light reflex during the initial three seconds of the experiment before stimuli presentation; (Fig. 2, lower left panel). In the remaining 117 s of the trial, as expected, visual detections induced small and repeated pupil dilations [4,42,12,32]. Our data show that: (i) dilations are steeper for Controls (Fig. 2, lower middle panel); (ii) Distractors evoked an inverse (constricting) pupillary response for both groups as expected but with a slightly but statistically significant greater amplitude observed in the Controls group. (Fig. 2, lower right panel); (iii) the overall pupil variability was larger for Controls (Fig. 3). These findings are consistent with challenges observed in ADHDs regarding the effective allocation (and disallocation) of attentional resources to the sequence of Targets (and Distractors). We provide below a neurobiological rationale which involves a role of Locus Coeruleus.

### 4.2. Locus coeruleus (LC), catecholaminergic activities and ADHD

Catecholamines, including dopamine and norepinephrine (i.e. noradrenaline), have a modulating role in perception and cognition with enhancing effects on perceptual acuity, decision making and the execution of goal-directed behavior[4,6]. More specifically, in situations of uncertainty, they have a role in consolidating and supporting attention and decision making[12]. The locus coeruleus (LC), a small nucleus located deep in the brainstem, is one of the primary sources of norepinephrine in the brain and strongly influences dopamine neural activity and dopaminergic receptors in general[29,26]. Dysfunction in the LC system has been implicated in the etiology of ADHD[3].

### 4.3. Locus coeruleus LC, catecholaminergic activities and the pupil

The size and dynamics of the pupil depend on a variety of sensory and attentional factors acting synergistically on the two muscles of the iris: the sphincter and the dilator. The Edinger–Westphal (EW) complex in the midbrain is the main parasympathetic nucleus of the pupil. It receives excitatory inputs from retina and accommodative centers; light, for example, constricts the sphincter, relaxes the dilator, and thus produces pupil constriction[22,44]. We can refer to this process as the “anti-attentional” pathway.

Baseline activity in the LC directly projects to the EW complex[23]) in an opposite inhibitory fashion causing pupil dilation[4,42,32,12]. The cortex itself exercises an influence on the pupil through widespread catecholaminergic inhibitory projections to the EW nuclei and, thus, directly mediates pupillary responses to cortical activity[19,44]; general arousal for example, or alertness, or sustained mental processing produce pupil dilation. [14,16,18,41,30]. The posterior hypothalamic nucleus is also closely interconnected with the LC[44,13]and produces enlargement of the pupil by direct activation of the dilator muscles[22]. We can use the term “attentional pathway” to refer to this constellation of processes.

### 4.4. Phasic and tonic imbalances of the dopamine system in ADHD

The neurobiology of ADHD is complex and heterogeneous; research has focused primarily on catecholaminergic deficiency, mostly dopamine, which are also the main target of pharmacological treatments, generally based on stimulant medications[9,43,1]. The impact of dopaminergic deficit on behavior and cognition can be summarized as an improper balance between phasic and tonic activities in the distributed catecholaminergic network including the LC and yielding: (i) inability of maintaining an appropriate arousal tone and sustained attention during focused tasks, (ii) ineffective initialization of salient-related phasic signaling for perceptual processing and preparation of responses[4,15,11,36,28,1] and (iii) altered suppression of ignored distractors likely linked to increased distractibility [8,35]. Elderly patients with cognitive decline show a similar impairment of distractor suppression in their fMRI brain measures[8].

### 4.5. Our findings regarding pupil behavior align with each of these three imbalances

Attentional and non-attentional processes engage in an agonist–antagonist interplay within the muscular apparatus of the iris sphincter and dilator. Attentional processes involve the sympathetic pathway, which, as discussed earlier, is responsible for modulating pupil size during active cognitive tasks and decision-making. It achieves this through direct mechanisms involving sympathetic hypothalamic innervation of the dilator muscle and indirect mechanisms involving cortical and locus coeruleus catecholaminergic projections on the Edinger-Westphal hub that innervates the sphincter muscle. The sympathetic attentional pathway is a dilator, and it measures attention. Conversely, the parasympathetic non-attentional pathway is involved in responding to passive and unattended stimuli, including light and general retina stimulation, irrespective of their visual significance. The parasympathetic non-attentional pathway is a constrictor, and it responds to passive perception[22].

In our experimental setup, the appearance of a patch in the visual field, even under isoluminant conditions, is expected to trigger a non-attentional parasympathetic pupil constriction [48,5]. In the case of attended Targets, this constriction is effectively counterbalanced by a strong attentional dilation, as depicted in Fig. 2 (lower middle panel). Non-attended Distractors cause constriction (Fig. 2, lower right panel); the difference in magnitude between the two groups suggests the existence of a counteracting attention-resistant and dilatory component within individuals with ADHD that slightly hinders their ability to exhibit the expected level of constriction in response to the non-attended stimuli.

### 4.6. Limitations, future directions and conclusion

Although some pupil variables, such as velocity and baseline diameter, are known to be attenuated by age, our cohort, which ranges from late teens to late thirties, appears to be unaffected by such age-related effects, as demonstrated in prior research (e.g., [34]. This is why we omitted to consider age in our analysis. Future experiments are recommended to characterize and compare the principles elucidated in this study with ADHD subjects that include childhood, pre-adolescents, and older populations.

The protocol introduced in this study is relatively straightforward to program and administer. The visual task is easily comprehensible and can be followed by individuals of all ages, requiring minimal cooperation from the subjects. The presentation display includes a central fixation point and two simple flickering sources of visual stimulation positioned randomly on either side of the fixation point, serving as targets or distractors of attention.

Both target and distractors can be repetitively displayed, enabling the collection of a substantial amount of data points for statistical analysis in a relatively short time. Multiple pupillary signatures have been demonstrated to be valuable for diagnostic purposes. In the case of targets, we look at the briskness of the corresponding pupil dilation, which tends to be slower in individuals with ADHD. For distractors, we look at the amplitude of the corresponding constriction, which is typically smaller in individuals with ADHD. Overall, individuals with ADHD exhibit smaller pupils and reduced variability during the trial.

Our findings align with those reported in a similar experiment conducted by Aboitiz’s group[46], which utilized a different type of visual stimuli and a younger population (10–11 years-old). To the best of our knowledge, this is the only other literature available on this subject. The two studies can be distinguished based on two specific aspects of the protocol. Firstly, the cognitive load involved in our task was limited to a simple target detection (as opposed to the requirement of memorizing a visual pattern that is embedded within a sequence containing other distracting images). Secondly, we introduced Distractors as distinct and independent perceptual events, allowing for a comparative analysis of their influence on the pupil response in comparison to that elicited by Targets. This allowed us to investigate how processes related to suppressing distractions are also involved in ADHD.

Both works provide a valuable foundation for future research aimed at developing and refining a psychophysics paradigm that can be used as a biomarker to improve ADHD evaluation and nosology. This research endeavor promises to not only enhance our understanding of ADHD’s underlying mechanisms but also to contribute significantly to the advancement of diagnostic tools and therapeutic interventions for individuals with ADHD. By building upon the insights and methodologies presented in these studies, the field can aim to develop more precise, efficient, and personalized approaches for the assessment and treatment of ADHD, ultimately benefiting both patients and the broader fields of neurology and psychiatry.

## Data Availability

The data that has been used is confidential.
